# Robustness of large-area suspended graphene under interaction with intense laser

**DOI:** 10.1038/s41598-022-06055-4

**Published:** 2022-02-16

**Authors:** Y. Kuramitsu, T. Minami, T. Hihara, K. Sakai, T. Nishimoto, S. Isayama, Y. T. Liao, K. T. Wu, W. Y. Woon, S. H. Chen, Y. L. Liu, S. M. He, C. Y. Su, M. Ota, S. Egashira, A. Morace, Y. Sakawa, Y. Abe, H. Habara, R. Kodama, L. N. K. Döhl, N. Woolsey, M. Koenig, H. S. Kumar, N. Ohnishi, M. Kanasaki, T. Asai, T. Yamauchi, K. Oda, Ko. Kondo, H. Kiriyama, Y. Fukuda

**Affiliations:** 1 Graduate School of Engineering, Osaka University, 2-1 Yamadaoka, Suita, Osaka 565-0871 Japan; 2 Institute of Laser Engineering, Osaka University, 2-6 Yamadaoka, Suita, Osaka 565-0871 Japan; 3 Department of Physics, National Central University, No. 300, Jhongda Rd., Jhongli, Taoyuan 320 Taiwan; 4 Interdisciplinary Graduate School of Engineering Sciences, Kyushu University, 6-1 Kasuga-Kohen, Kasuga, Fukuoka 816-8580 Japan; 5 Molecular Science and Technology, Taiwan International Graduate Program, Academia Sinica and National Central University, No. 1, Roosevelt Rd., Sec. 4, Taipei, 10617 Taiwan; 6 Institute of Space and Plasma Sciences, National Cheng Kung University, 1 University Road, Tainan City, 70101 Taiwan; 7 Graduate Institute of Energy Engineering, National Central University, No. 300, Jhongda Rd., Jhongli, Taoyuan 320 Taiwan; 8 Department of Physics, York Plasma Institute, University of York, York, YO10 5DD UK; 9 Glen Eastman Energy b.v., Dockwards 1, Waalhaven O.z. 83M, 3087 BM Rotterdam, The Netherlands; 10 LULI, CNRS, CEA, Ecole Polytechnique,UPMC, Univ Paris 06: Sorbonne Universites, Institut Polytechnique de Paris, 91128 Palaiseau cedex, France; 11 Department of Aerospace Engineering, Tohoku University, 6-6-01 Aramakiazaaoba, Aoba-ku, Sendai, 980-8579 Japan; 12 Graduate School of Maritime Sciences, Kobe University, Kobe, 658-0022 Japan; 13 Kansai Photon Science Institute (KPSI), National Institutes for Quantum Science and Technology (QST), 8-1-7 Umemidai, Kizugawa, Kyoto 619-0215 Japan

**Keywords:** Laser-produced plasmas, Photonic devices, Two-dimensional materials

## Abstract

Graphene is known as an atomically thin, transparent, highly electrically and thermally conductive, light-weight, and the strongest 2D material. We investigate disruptive application of graphene as a target of laser-driven ion acceleration. We develop large-area suspended graphene (LSG) and by transferring graphene layer by layer we control the thickness with precision down to a single atomic layer. Direct irradiations of the LSG targets generate MeV protons and carbons from sub-relativistic to relativistic laser intensities from low contrast to high contrast conditions without plasma mirror, evidently showing the durability of graphene.

## Introduction

Laser driven ion sources have been widely investigated for pure science, plasma diagnostics, medical and engineering applications^1,2^. Recent developments of laser technologies allow us to access radiation regime^3–5^ of laser ion acceleration with relatively thin targets^6–11^. However, the thinner target is the less durable and can be easily broken by the pedestal or prepulse through impact and heating prior to the main laser arrival^12,13^. One of the solutions to avoid this is plasma mirror, which is a surface plasma created by the foot of the laser pulse on an optically transparent material working as an effective mirror only for the main laser. So far, the ion acceleration in extremely thin target regime ($< 10\ \hbox {nm}$) has been investigated with plasma mirrors^6^, and it is necessary to use plasma mirrors even in moderately thin target regime (10–100 nm) to realize energetic ion generation^7–11^. The combinations of relatively thin targets and plasma mirrors have been successful and the proton energies are approaching 100 MeV^9^. However, it is costly to make thin and flat targets using conventional 3D materials, and installing and operating plasma mirrors at high repetition rate is also costly.

We have developed large-area suspended graphene (LSG) for proton radiography intended to use laboratory astrophysics experiments with a relatively small laser facility, where no plasma mirror is equipped^14,15^. In such small laser facilities, the radiations from the laser matter interactions by shooting $\upmu$m thick solid targets would be a serious problem. Although $\upmu$m thick solid targets can generate energetic ions with large laser facilities^12,16^, it is not practical for such small laser facilities. Constructing the radiation shielding is also practical unavoidable issue since the massive lead normally used for radiation shield might exceed the acceptable floor strength in such small facilities. We have to suppress the radiation itself by reducing the number of encounters of relativistic electrons with atomic nuclei in the target. To this end we have to develop a thin and strong, low Z material, reasonable to fabricate, that is, graphene. Graphene is stronger than diamond at extremely thin regime^17^, and much more reasonable for mass-production. Graphene has several unique features suitable for laser ion acceleration, i.e., the thinnest, lightest, transparent and the strongest material at this regime^18^. Direct irradiations of the LSG targets from non-relativistic to relativistic laser intensities without plasma mirror generating MeV carbons and protons evidently show the durability of graphene. We further demonstrate the durability of graphene with low contrast laser condition with $10^{-6}$ prepulse with respect to the peak intensity. Our double-layer LSG corresponding to 2 nm thick is the thinnest target generated MeV protons even without plasma mirror. We also conduct relevant particle-in-cell (PIC) simulations, indicating that although the graphene is melted prior to the main laser peak due to the prepulse and pedestal, the target plasma keeps overcritical density until the main pulse arrival. Our results show the robustness MeV ion acceleration by direct irradiation with LSG. This can open and extend the laser-driven ion acceleration and its applications to the rest of laser facilities with moderate laser intensities and no plasma mirror equipped.

## Results

### Large-area suspended graphene (LSG)

**Figure 1:**
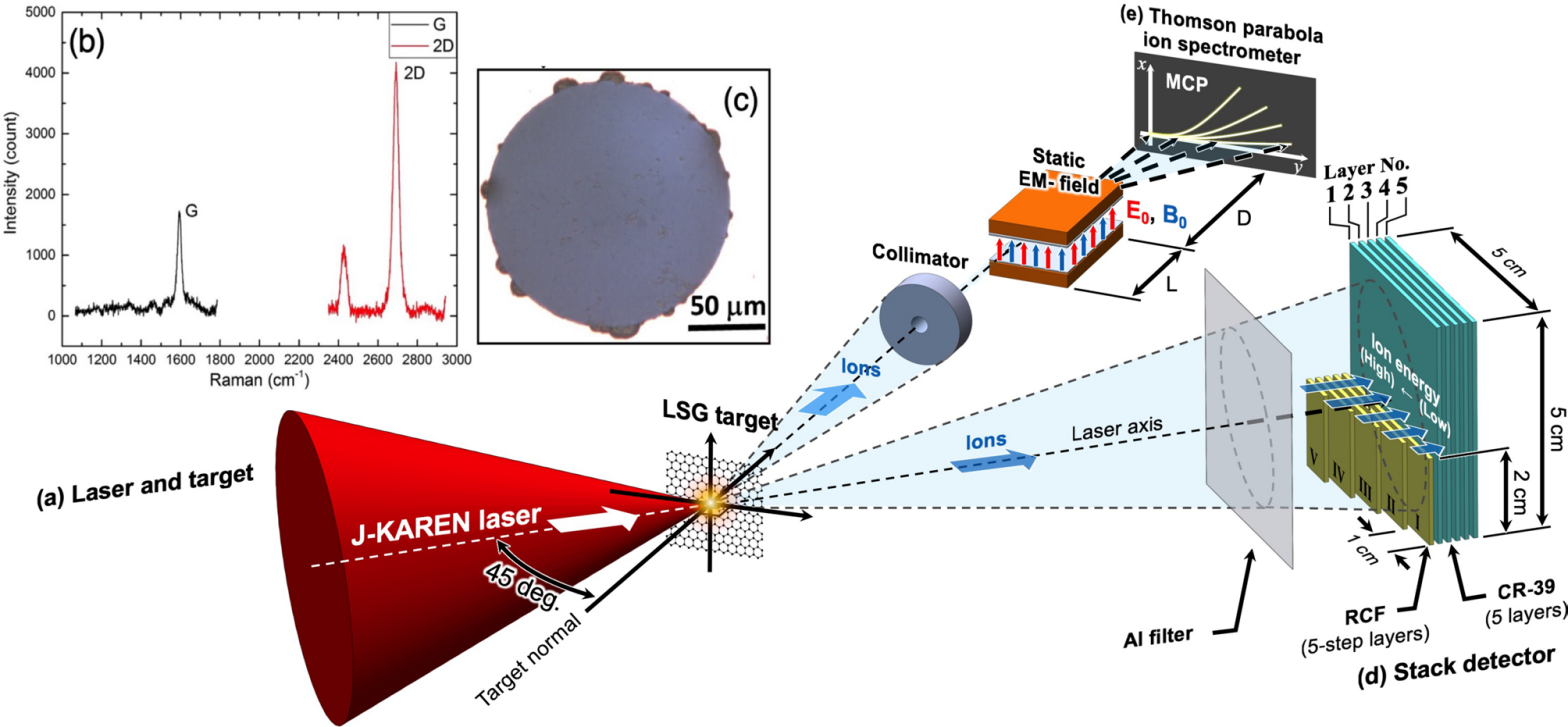
(**a**) Schematic setup of the experiment with the large-area suspended graphene target (LSG). (**b**,**c**) The Raman spectrum and the optical microscope image for a typical LSG, respectively. The accelerated ions are detected with: (**d**) a stack of radiochromic films (RCFs) and solid state nuclear track detector (CR-39) and (**e**) Thomson parabola spectrometer.

The experiments are performed with the J-KAREN-P laser at the short-F chamber, Kansai Photon Science Institute in Japan^19^. Figure 1a shows the schematic images of experimental setup and configuration of diagnostics. The details are written in “Method”. Figure 1b,c show the typical Raman spectrum and image with optical microscope respectively, from which the narrow bandwidth of 2D band ($< 30$ cm$^{-1}$) and the higher intensity ratio ($> 2.5$) of the 2D and G bands (I(2D)/I(G)) confirmed the presence of single-layer LSG^20,21^. The small peak next to the 2D peak comes from the tiny curvature of LSG due to the large aspect ration of LSG. Note that the ideal LSG is 0.34 nm, while the obtained transferring graphene is close to 1 nm due to molecular adsorption on the surface^15^. By transferring graphene layer by layer, we control the target thickness at 1 nm accuracy^15^.

By irradiating the LSGs with the intense laser, energetic ion beams are produced. The ion diagnostics are stack detector composed of radiochromic films (RCF, GAFCHROMIC XR-RV3) and solid state nuclear track detectors (CR-39, HARZLAS TD-1, 0.9 mm) as shown in Fig. 1d, where the higher energy ions can penetrate through the deeper, and Thomson parabola spectrometer (TPS) as in Fig. 1e. When a solid target is irradiated with an intense laser, a proton beam is generated independent of target material due to the surface contamination from moisture in the air. The CR-39s allow us to distinguish heavier ions or mostly carbons from protons by the size of ion pits. Furthermore, TPS provides charge-to-mass ratio together with the energy spectra. Further details of TPS analyses and the stack detector are provided in the Supplementary information. We have two series of experiments with a relatively high contrast and with a low contrast conditions. In the former case we have tested defocused non-relativistic laser intensity and the best focus relativistic intensity.

**Figure 2:**
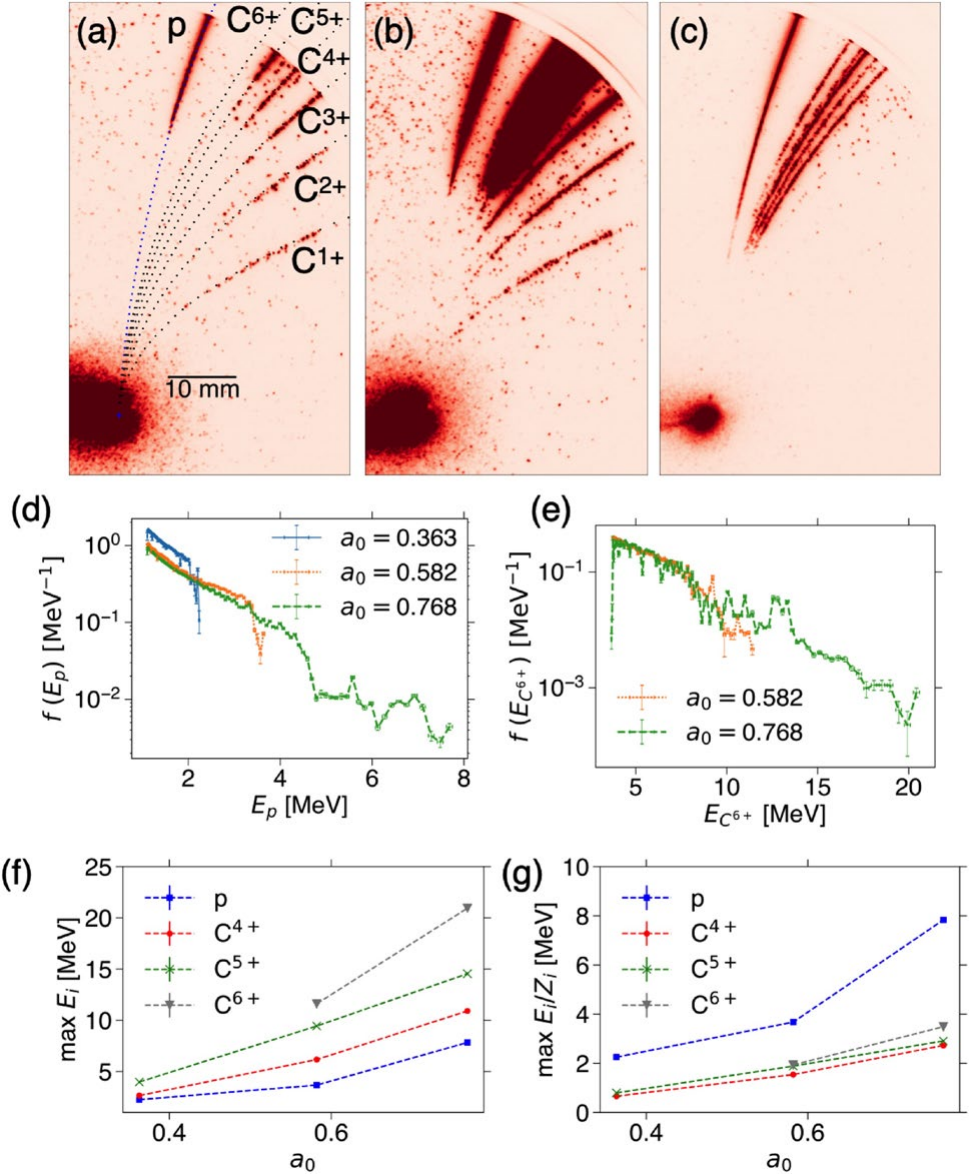
High contrast defocused shots: energy dependence. TPS images from three successive 4L-LSG target shots with increasing laser energies with (**a**) 4.40 J measured before the compression chamber, corresponding intensity $I = 2.78 \times 10^{17}$ W cm$^{-2}$, where the 32% enclosed energy is taken into account^22^, and the normalized intensity $a_0 = 0.363$, (**b**) 11.3 J, $7.15 \times 10^{17}$ W cm$^{-2}$, $a_0 = 0.582$, and (**c**) 19.7 J, $1.25 \times 10^{18}$ Wc m$^{-2}$, $a_0 = 0.768$. (**d**,**e**) The energy distribution functions of protons and C$^{6+}$ after subtracting the background signals as discussed in the Supplementary information. (**f**,**g**) The maximum energies of protons and carbons (C$^{4+}$–C$^{6+}$), and the maximum energies divided by the charge state *Z*, respectively.

### High contrast defocused shots

Figure 2 shows the TPS images from three successive target shots with the same target thickness of 4 nm but different laser energies. Figure 2a corresponds to the lowest intensity shot among all the shots in the two experimental campaigns. Using LSG, even with sub-relativistic laser pulse can produce MeV protons and carbons. The energy distribution functions are evaluated as in Supplementary Figure 1. Note that as shown in Supplementary Figure 2, there are also about 30% oxygen ions. The oxygen ion energy tends to be lower than carbon energy as well as the number. While for the lower intensity shots in Fig. 2a,b, the lower Z carbons (C$^{1+}$ and C$^{2+}$) are recognized, for the higher intensity shot in Fig. 2c they are not clear. In contrast, higher *Z* carbons (C$^{6+}$) are not recognized in Fig. 2a. The proton and carbon energies are the higher for the higher laser energy at sub-relativistic intensity as in Figs.2d,e. Note that the MCP and phosphor voltages are higher for Fig. 2a,b; the signal level is too high so that the carbon and oxygen lines are overlapped, and thus we reduce the voltages for higher energy shot in Fig. 2c. This is nothing to do with the evaluation of ion energy, but just relevant to the saturation level seen in lower energy part of the distribution functions as discussed in Supplementary Figure 1.

When the protons and carbons are accelerated by the same potential field, which is most of the case in laser ion acceleration, the carbon energy is *Z* times larger than that of proton due to the difference of charge-to-mass ratio as,

1 $$\begin{aligned} \frac{1}{2}m_iv_i^2=Z_i e\phi , \end{aligned}$$

where $m_i$ is the ion mass, $v_i$ is the ion velocity, $Z_i$ is the charge state, *e* is the element charge, and $\phi$ is the electric potential accelerating the ions. Figure 2f shows the maximum ion energies $E_i \equiv mv_i^2/2$ in terms of the normalized intensity $a_0$, where the higher intensity results in the higher ion energy. The higher *Z* tends to have the higher energy as expected by Eq. (1). As seen in Fig. 2g, while the carbon ions with different charge states have similar values in $E_i/Z_i$, the proton energy is higher than that of carbon. This will be discussed later with particle-in-cell (PIC) simulations.

We have also tested the different thickness of LSGs. The thickness dependence is shown in Supplementary Figure 3. At the defocused non-relativistic intensity, the optimal target thickness for radiation pressure acceleration (RPA) ranges from a few nm to $\sim$ 30 nm^23^. Although there are not many shots, the thickness dependence at this regime seems weak.

**Figure 3:**
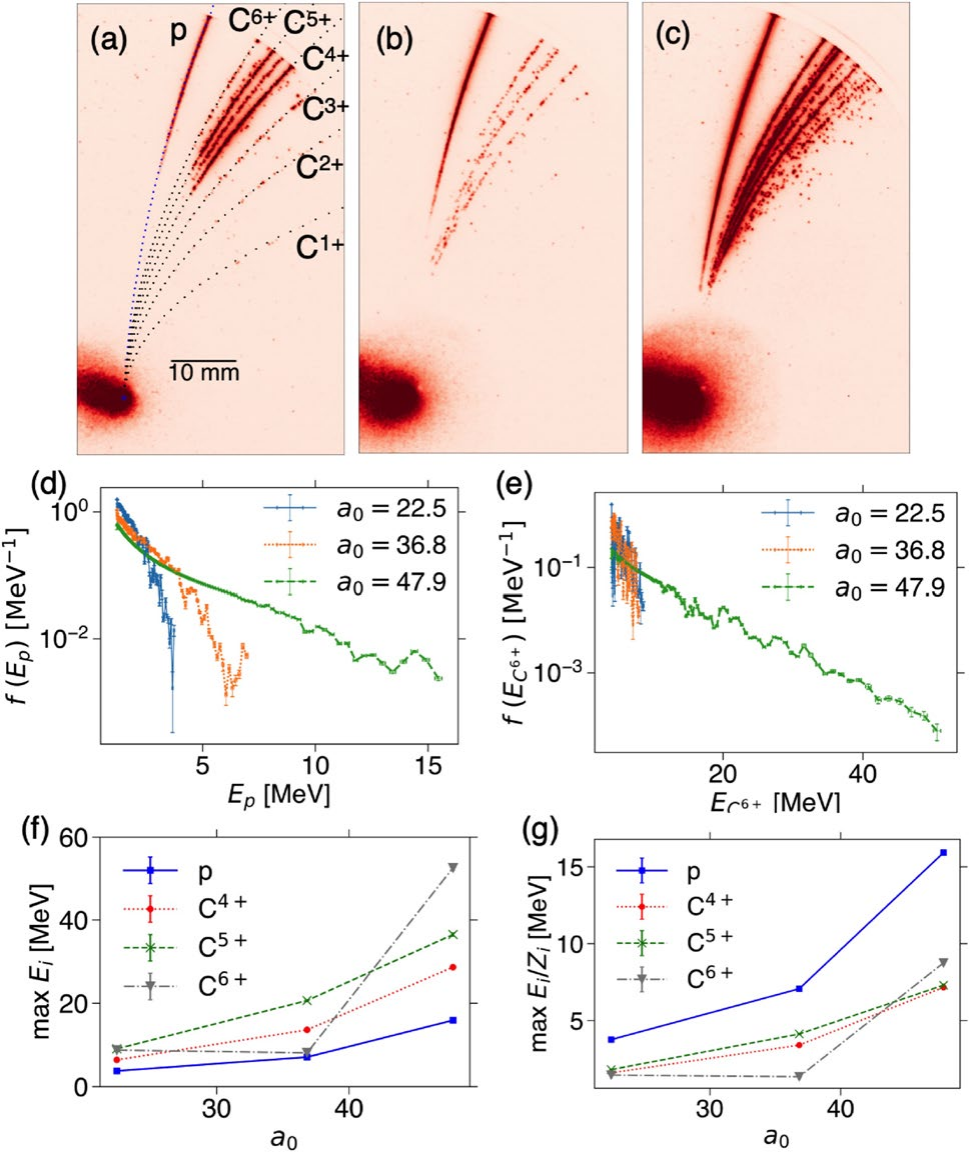
High contrast best focus shots: energy dependence. Same as Fig. 2 except for 8-layer LSG with best focus: (**a**) 4.24 J, $I = 1.06 \times 10^{21}$ W cm$^{-2}$, $a_0 = 22.5$, (**b**) 11.4 J, 2.86 $\times 10^{21}$ W cm$^{-2}$, $a_0 = 36.8$, and (**c**) 19.3 J, 4.83 $\times 10^{21}$ W cm$^{-2}$, $a_0 = 47.9$.

### High contrast best focus shots

Figure 3 shows the same plots as Fig. 2 except for 8-layer LSG, i.e., 8 nm thick targets at the best focus. By comparing Fig. 3a–c with Fig. 2a–c, while overall low *Z* signals are weaker for the best focus shots, it is consistent that the higher laser energy and intensity results in the higher ion energies. Note that while Fig. 2 is 4-layer LSG, Fig. 3 corresponds to 8-layer LSG. However, as shown in Supplementary Figure 3, at least at the non-relativistic intensity the thickness dependence on the ion acceleration is weak. The energy distribution functions in Fig. 3d,e show about twice higher energies than those of defocused shots in Fig. 2d,e. In Fig. 3f,g the maximum C$^{6+}$ energy for the middle intensity, corresponding to Fig. 3b, shows lower energy than those of C$^{4+}$ and C$^{5+}$. We estimate the maximum energies once the standard deviation exceeds the mean, and thus, it is hard to precisely determine the maximum energy for weak disconnected signals as in Fig. 3b. At the best focus, the intensity is orders of magnitude higher than that of the defocused shots, however, the proton and carbon energies are just about twice higher than the defocused cases. This indicates that the target thickness of 8 nm is too thin at $I \sim 5 \times 10^{21}$ W cm$^{-2}$. The optimum thickness can be $a_0$ times thicker than the values discussed in the defocus case.

**Figure 4:**
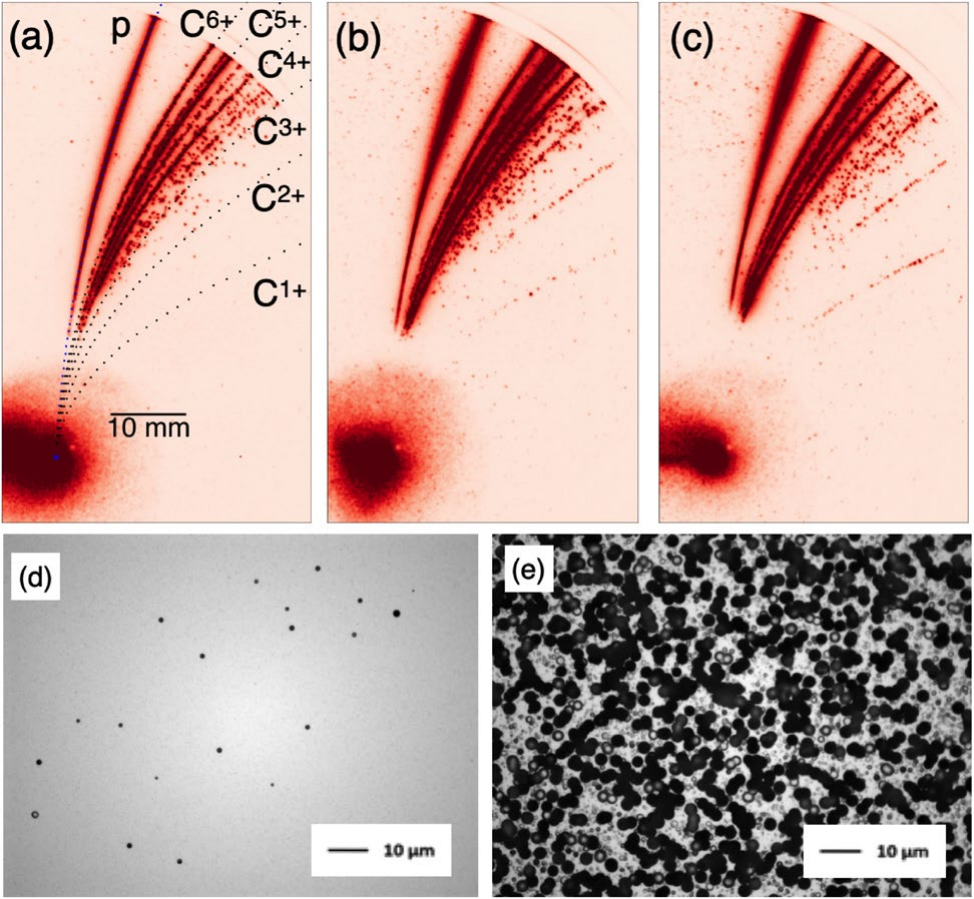
High contrast best focus shots: reproducibility. Three successive nominally identical shots with 8-layer LSG: (**a**) the same shot as in Fig. 3c, 19.3 J, 4.83 $\times 10^{21}$ W cm$^{-2}$, $a_0 = 47.9$, (**b**) 18.2 J, 4.55 $\times 10^{21}$ W cm$^{-2}$, $a_0=46.6$, and (**c**) 19.2 J, $4.81 \times 10^{21}$ W cm$^{-2}$, $a_0=47.8$. The ion pits on CR-39 in stack detector: (**d**) the proton pits on the second CR-39 with the aluminum foil and two RCFs, corresponding to the energy of 12.2–13.2 MeV, and (**e**) the carbon pits on the first CR-39 covered with a 12 $\upmu$m aluminum foil with the energy range between 14 and 94 MeV.

Figure 4a–c shows the TPS images from three successive nominally identical shots with 8-layer LSG at the best focus with laser energy $\sim$ 20 J. Note that Fig. 4a is the same shot as in Fig. 3c. Although Fig. 4c shows slightly lower energy, all the shots show similar signals. Figure 4d,e show the etched pits of protons and mostly carbons, respectively. The details on the stack detector are found in Supplementary information and the proton and carbon stopping energies are listed in Supplementary Table I and II, respectively. The stack detector accumulates all the 8-layer LSG shots including Figs. 3 and 4. As shown in Fig. 3 the higher laser energy results in the higher ion energies, and from the proton energy distribution functions in Fig. 3d, the highest energy proton pits observed in the stack detector (12.2–13.2 MeV) in Fig. 4d come from the highest intensity shots of Fig. 4a–c. The proton energy observed with stack detector is slightly lower than that of TPS. This is due to the detecting angle of each detector. We will discuss this with PIC simulation later.

Although the energy resolution of carbon with the stack detector is large at low carbon energy (14–94 MeV), the carbon energies measured with the TPS in Fig. 3e, where the maximum carbon energy $\sim 60$ MeV, is consistent with the stack result. From Fig. 3f, the carbon pits in Fig. 4e come not only from the higher intensity shots in Fig. 4a–c, but also from the middle intensity shot in Fig. 3b. Although It is not trivial to count the number of carbon pits in Fig. 4e since there are many ions and the pits overlap each other, the number of carbon pits in 10 $\upmu$m square region is typically $\sim$ 10. From this we can estimate how many carbons from the LSG accelerated as follows. The solid angle of the 10 $\mu$m square region of the stack detector located 157 cm away from the laser focal spot is $\sim (10^{-3})^2/157^2=4.06\times 10^{-11}$ sr, and thus, $10/(4.06\times 10^{-11})=2.46\times 10^{11}$ atoms/sr. As shown in Fig. 1, the TPS and the stack detector are located with an angle of 45 degrees. The ions are accelerated over the angle at least 45 degrees, and the solid angle for the cone with 45/2$^{\circ }$ is 0.478 sr. The number of carbon ions within the cone is $2.46\times 10^{11} \times 0.478=1.77 \times 10^{11}$. The areal carbon density of graphene is $\sigma = 3.82\times 10^{15}$ cm$^{-2}$, and assuming the accelerated graphene area of $\pi r^2$, for 4 shots of 8-layer LSG, $32 \times \pi r^2 \times \sigma =1.77 \times 10^{11}$. Therefore, $r=6.79$ $\mu$m in order to account for the number of observed carbon pits. In reality there are also oxygen ions, which tend to reduce the radius, and some of the ions go to the opposite direction as shown later with PIC simulation, which tends to increase the radius. As shown later with the PIC simulation, the carbons over 10 $\upmu$m are accelerated by the laser with 2 $\upmu$m spot size. This is consistent with the above estimation, and indicating all the carbon ions around the focal spot are accelerated.

**Figure 5:**
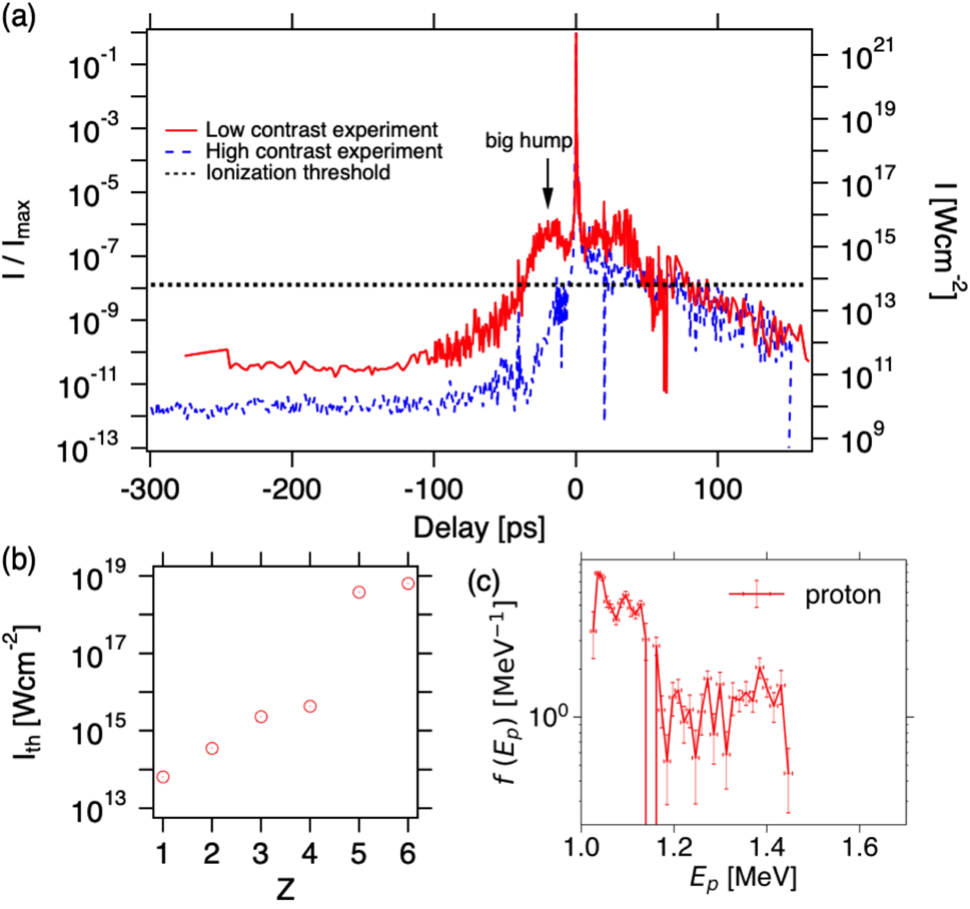
Low contrast shot with double-layer LSG. (**a**) Contrast measurements for low and high contrast experiments. The left and right axes show the normalized and maximum intensity being $I_{max}=5 \times 10^{21}$ W cm$^{-2}$, respectively. The horizontal dotted line represents the first ionization threshold of carbon for the field ionization. (**b**) The carbon ionization thresholds for each charge state. (**c**) The proton energy distribution function obtained from TPS with double-layer LSG. The laser energy is 14.8 J corresponding to $I= 3.71 \times 10^{21}$ W cm$^{-2}$ and $a_0=41.9$.

### Low contrast experiment

Figure 5a shows the contrast measurement for the low (high) contrast experiment with solid (dashed) line. The high contrast data is the same as in^19^. For reference, we also plot the first ionization threshold of carbon assuming the field ionization by laser^24^. The carbon ionization potential is obtained from the NIST Atomic Spectra Database^25^, and is plotted against charge state in Fig. 5b by converting the threshold intensities under assumption of the field ionization^24^. We do not consider here the graphene structure but just carbon atom. Nevertheless, the graphene will be destroy even one electron is kicked out by laser. In the low contrast experiment, there is a big hump of the contrast level $\sim 10^{-6}$ before $\sim 20$ ps of the peak intensity, and the before $\sim 40$ ps the foot of the hump exceeds the first ionization threshold represented with the dotted horizontal line in Fig. 5a. On the other hand, in the high contrast case, the small peak at $t=-13.6$ ps once exceed the threshold. The multi-layer LSG may survive, as we discuss later on the transmission and absorption, after the small prepulse until the rising foot of the main peak exceeding the threshold at $t=-5$ ps. The big prepulse in the low contrast experiment is very severe condition for extremely thin target regime. Therefore, this is the best condition to demonstrate the LSG durability.

Figure 5c shows the distribution functions with double-layer LSG corresponding to 2 nm thickness with the low contrast laser condition. As mentioned above, the pre-pulse and pedestal are major practical problem in the extremely thin target regime and the recent experiment^6–10^ all utilize the single or double plasma mirrors to suppress the pre-pulse and pedestal to realize the energetic ion acceleration. Even with such the large pre-pulse, the double-layer LSG generates MeV protons without plasma mirror. The 2 nm thick target is the thinnest target ever generated MeV protons; it is hard make 3D material as thin as this.

The low contrast shots with single-digit-nanometer thick LSGs are unstable; some show just proton as in Fig. 5c, and some show carbons as well shot by shot. However, the thicker targets, for instance 32-layer LSGs, show stable ion acceleration as in Supplementary Figure 4, even in the presence of the large pre-pulse as in Fig. 5a.

**Figure 6:**
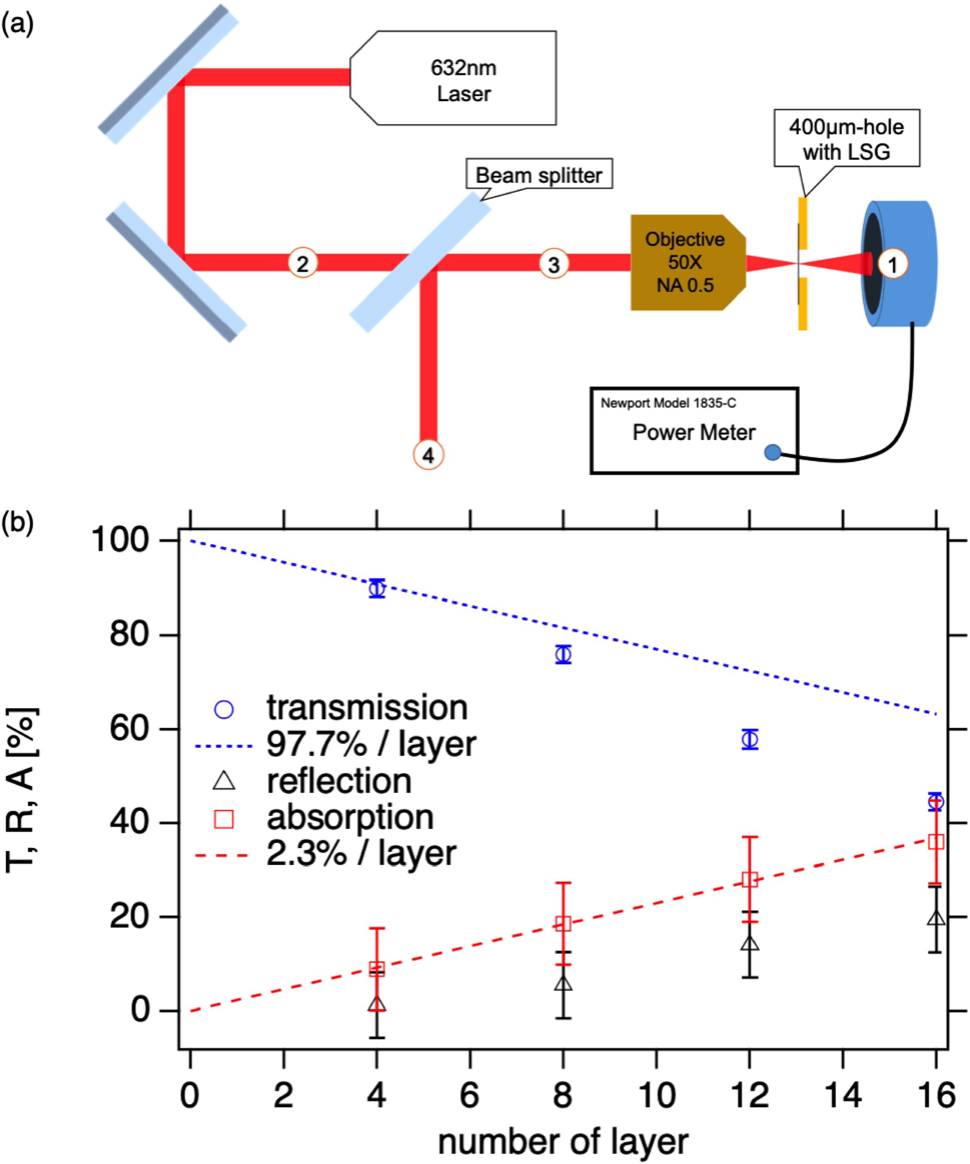
Off-line experiment: transmission and absorption. (**a**) Schematic image of setup for the LSG transmission/reflection measurement. (**b**) The transmission (*T*), reflection (*R*), and absorption rates (*A*) are plotted against the number of layer of LSG. The error bars represent the standard deviation of the measurements. The dotted lines represent the theoretical transmission (blue) and absorption rates (red)^26^.

### Transmission and absorption of LSG

Figure 6a,b show the schematic setup for the transmission/reflection measurements with weak laser and the results, respectively. We use a He–Ne laser with wavelength of 632 nm and focus the beam with an objective lens with the numerical aperture of 0.5 on to the graphene suspended over 400 $\upmu$m hole in atmospheric pressure. The wavelength dependence on transmittance of 632 nm light and 800 nm of J-KAREN is negligibly weak^26^. Measuring the laser powers at the position 1, the transmission rate *T* can be calculated by dividing the results with and without graphene on the hole, and thus, most reliable and the error is also small as shown in Fig. 6b. To acquire accurate reflection rate *R*, the optical properties of the beam splitter ($T_s, R_s$) and the lens ($T_l, R_l$) are obtained in advance with the focused beam passing through an empty hole. Provided the laser power measured at position 2 is *P*, the laser power measured at position 4 is $P T_s T_l^2 R R_s + P T_s R_l R_s$, from which *R* can be derived. As the reflection power is weak, the error bars on the reflectivity is large. We measure the transmission (*T*) and reflection rates (*R*), and obtain the absorption rate (*A*) by $A=100-(T+R)$.

It is known that a single atomic layer graphene absorbs white light $A = 2.3\%$ defined as $\pi \alpha \sim 2.3$%^27^, where $\alpha =1/137$ is the fine structure constant, and that the graphene is highly transparent ($T\sim 97.7\%$) and the reflectivity is negligibly small ($R < 0.1\%$)^26,28^. We plot the 97.7% transmission (dotted line) and 2.3% absorption (dashed line) per layer for reference in Fig. 6b. With 4-layer LSG, the transmission is comparable to the ideal value of 97.7% transmission per layer. The transmission deviates from the theoretical line as the number of layer increases. The reflection rate also increases as the number of layer increases; it is not negligible for many layer LSGs. However, the measured absorption rate excellently agrees with the theoretical line of 2.3% absorption per layer. This indicates that the contaminants reflect the light but not absorb it at low intensity light. As indicated in Supplementary Figure 2, the LSG contains the contaminant. There are two significant outcome of LSG when used as targets for laser-driven ion sources; (1) the light exerts pressure only on the contaminants until the LSG is ionized, and (2) most of the laser power (97.7%) is not absorbed by LSG until the LSG is ionized, where only 2.3% laser power is absorbed and can convert to heat causing the LSG melting prior to the main laser peak. Furthermore, the thermal conductivity of graphene is extremely high^29^, and thus, the heat can diffuse quicker than other materials within the layer, but not for interlayers. As discussed above, the high contrast measurement in Fig. 5a shows a small peak at $t=-13.6$ ps exceeding the ionization threshold. For instance, the 8th layer graphene in 8-layer LSG, the pre-pulse and pedestal are $2.3\times 7$= 16.1% less intense. The last graphene may not be destroyed by the small pre-pulse. These significant features make the LSG durable against the pre-pulse and pedestal as shown above both for high contrast and low contrast experiments.

**Figure 7:**
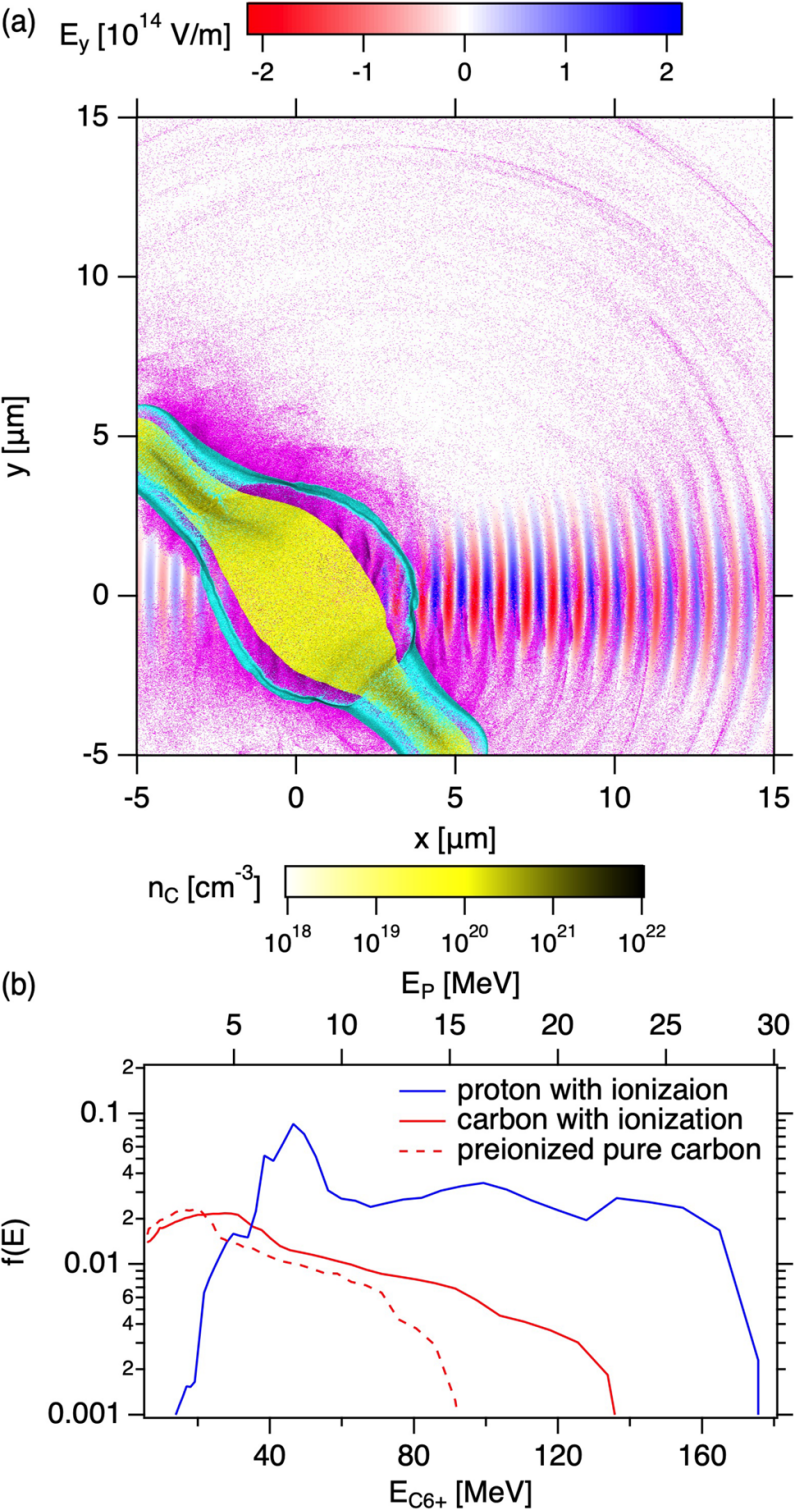
(**a**) The snapshots from 2D Particle-in-cell (PIC) simulation at $\sim$ 20 fs from the laser peak arrival at the 8 layer LSG target for best focus. The laser electric field $E_y$ (blue-red), electron $n_e$ (magenta), carbon $n_C$ (yellow), and proton number densities $n_p$ (cyan) are overlaid. The color scales for the number densities are set to be identical. (**b**) Energy distribution functions calculated from all the protons (blue) and carbons C$^{6+}$ (red) in the simulation box of (**a**), normalized as $\int f dE=1$. The top and bottom axes show the proton and carbon energy, where the top axis is adjusted as 1/6 of carbon energy. The red dashed line represents the pure carbon with pre-ionization.

### Two dimensional particle-in-cell simulations

We perform 2D particle-in-cell (PIC) simulations with an open code EPOCH^30^. The simulation details are given in “Method”. Figure 7a shows the snap shot of 2D simulation for best focus shot with 8-layer LSG at 20 fs from the laser peak arrival at the target with the target ionization by laser. The laser propagates from the left to the right along the *x* direction. The laser pedestal and pre-pulse are not taken into account. We estimate the total number of electrons from LSG including water contaminations, and then replace the oxygen with carbon for simplicity; the major component of heavy ions are essentially from graphene carbons as shown in Supplementary Fig. 2b. In Fig. 7a, the lightest electrons (magenta) are firstly accelerated by the laser electric field (red-white-blue), and then, the second lightest protons (cyan) follow due to the space charge effect. The protons are localized in thin layers even though the protons distribute randomly in space within the target and mixed with carbons in the initial condition. This indicates that the heavier carbons assist to accelerate the lighter protons. The protons and carbons expand or are accelerated in the target normal direction rather than the laser propagation direction. This is true for the space charge acceleration and also for the RPA by considering the momentum conservation when the laser is specularly reflected by the target plasma. Note that the simulation domain is limited around the interaction region, the most of electrons are removed from the carbon. In simulations we simply consider C$^{6+}$. Figure 7b shows the energy distribution functions of protons and carbon (C$^{6+}$), where top and bottom axes show the proton and carbon energy, respectively, and we set the bottom energy range to be 6 times of the top axis. The proton energy is comparable but higher than the 1/6 of carbon energy as in Fig. 7b, as discussed above the protons are preferentialy accelerated with the aid of carbons, which is consistent with the experimental results in Fig. 3g. Note that in the experiment TPS observes the limited solid angle. In contrast, in the simulations we integrate the ions over the simulation box. The ions can be accelerated or decelerated, and can be scattered in terms of angle, when they propagate to the TPS. These can be different from the simulations.

The numerical obtained maximum ion energies are factor two larger than those in the experimental results in Fig. 3d,e for the highest intensity shot. This indicates that the target is ionized before the main laser peak arrival in the experiment due to the pre-pulse and pedestal. Note that it is already significant for nm thick graphene target without plasma mirror to produce MeV protons and carbons; other materials cannot produce energetic ions without plasma mirror at this thickness. The contrast measurement for the high contrast condition also suggests the target ionization 5–10 ps prior to the main laser peak in Fig. 5a. Another reason for the overestimation of numerical ion energies is the dimensionality of PIC simulations. The space charge is strongly depending on the dimensionality, i.e., the lower dimension overestimate the space charge electric field^31–33^.

Another possibility account for the lower experimental results is that the LSG optical properties show in Fig. 6. We consider the homogeneous expansion including the electrons from the contaminants, however, as discussed above, the weak light pressure before the ionization of graphene can act only on the contaminant. Therefore, we simply consider the pure graphene density with pre-ionized expanded target by factor 10 with the reduced density. As shown in Supplementary Figure 5, the ionization is not essential and by assuming the pre-ionization of the targets due to the pre-pulse and pedestal, the expanded targets keeping the total number of particles same result in smaller ion energies as in the experiment. The carbon energy is still slightly higher than but comparable to the experimental energies as shown with the dashed line in Fig. 7b.

## Summary and discussions

We have developed an extremely thin target, large-area suspended graphene (LSG)^15^, where graphene is the thinnest, lightest, transparent, and strongest 2D material^18^. By transferring graphene layer by layer, we control the target thickness by 1 nm accuracy. We measured the LSG transmission, reflection, and absorption rates, where the transmission and reflection deviate from the theoretical prediction, however, the absorption rate is nearly identical to the theoretical expectation of 2.3% per layer, which is determined from the fine structure constant^27^. By irradiating the LSGs with J-KAREN laser from non-relativistic to relativistic intensities with high and low contrast conditions without plasma mirror, we successfully observe the MeV protons and carbons. With the high contrast condition, we have 16 effective shots in total and the MeV protons and carbons are stably generated in all the shots as shown in Figs. 2, 3 and 4, and Supplementary Figure 3. The double-layer LSG is the thinnest target that has produced energetic ions with intense lasers, even without plasma mirror with low contrast condition. The number of carbons estimated with the stack detector shows extremely efficient acceleration where most of the graphene carbons interacting with laser are accelerated. Even with the large pre-pulse in the low contrast experiment, MeV protons and carbons are stably generated by shooting thicker targets as in Supplementary Figure 4.

We also perform 2D PIC simulations with various conditions. Although the target seems ionized prior to the main laser peak, the factor two difference from the ideal simulation without considering any pre-pulse and pedestal is already significant; there is no experimental report on the energetic ion production without plasma mirror at a few nanometer thickness with other materials. This clearly shows that, even though the graphene is melted by pre-pulse, it can keep the critical density until the main laser arrival without plasma mirror. In order to understand the interactions between graphene and pre-pulse, we need molecular dynamic simulation with time dependent density functional theory, where the unique graphene properties taken into account, for instance, the absorption property of 2.3% determined with the fine structure constant and high thermal and electrical conductivities within a layer but not inter layers, together with the PIC simulations. Note that there is no comprehensive numerical code that includes the ab-initio quantum theory together with the corrective plasma dynamics. Our results imply the necessity of such code. This will be discussed elsewhere in the future.

In PIC simulations, unless we use a code taking the molecular and atomic structures properly included such as the time-dependent density functional theory based molecular dynamic code, there is no difference between amorphous carbon and graphene. We take into account the ionization of carbon atoms in Fig. 7a, but there is no molecular structure of graphene. Suppose that amorphous carbon can be a few nanometer but cannot generate energetic protons and carbons without plasma mirror at this thin regime, then, PIC simulation without considering the pre-pulse and pedestal is meaningless since in reality there is no energetic ions. In terms of graphene, nevertheless, the MeV protons and carbons are generated without plasma mirror in reality, and the PIC simulations without considering the pre-pulse and pedestal results in about factor 2 overestimation. This is still useful information when one needs proton beam for applications, such as proton radiography. One can estimate the proton energy for radiography with PIC simulations by taking account the overestimation of factor 2.

Our experimental and numerical results show that the target thickness is still too thin, and also that we do not need that high laser intensity to produce several MeV ions. As shown in Fig. 2 the defocused low energy J-KAREN-P laser at $\sim 10^{17}$ W cm$^{-2}$ can generate several MeV protons and carbons; table-top lasers may be also used to generate MeV protons and carbons at high repetition rates with LSGs. Large graphene sheets can be made in industries and we can transfer them to the substrates with many holes. We can use a rotational stage to shoot the suspended graphene at high repetition rate in the future. Our results also indicates that the thicker LSG with plasma mirror may be used for exploring the energy frontier of laser-driven energetic ions. Finally, nanometer thin but strong LSG can be used to mount other targets, such as nanostructure targets, which cannot stand by themselves. These will be explored in the future.

## Method

### J-KAREN-P laser

The J-KAREN-P laser is focused with an F/1.3 off-axis parabolic mirror (OAP) on the LSG without plasma mirror. The pulse energy, duration, focal spot, and the intensity are $\sim$ 10 J on target, 40 fs, $\sim 2 \upmu$m, and $\sim 5 \times 10^{21}$ W cm$^{-2}$, respectively, measured just before the experiments. We have two series of experiments: the first one with high contrast at 45$^{\circ }$ laser incidence to the target normal direction to avoid the back reflection of incident light to destroy the upstream optics as in Fig. 1, and the second is low contrast experiment with 10$^{\circ }$ laser incidence. The diagnostics are common for both cases. Since the geometry of the tight focus laser with F/1.3, a hole on a substrate to suspend graphene has to be large enough not to irradiate the substrate as shown in Supplementary Fig. 6. We have to use 400 $\upmu$m hole for safety with the 45$^{\circ }$ incidence. The success rate to make the single and double-layer graphene suspended over 400 $\upmu$m hole is still low, and we use 200 $\upmu$m hole for double-layer LSG.

### 2D particle-in-cell (PIC) simulations

We perform 2D PIC simulations for the J-KAREN-P experiment using an open simulation code EPOCH^30^. We fix the laser parameters based on the experimental conditions, and linearly polarized Gaussian beam in simulation plane. We consider the target ionization with the laser electric field via barrier suppression, tunneling, and multi photon ionization processes with a standard option of EPOCH. Ionization thresholds of atoms are cited from Atomic Spectra Database^25^.

The areal density of graphene is $\sigma = 3.82\times 10^{15}$ cm$^{-2}$. Our LSG thickness is 1 nm / layer, and thus, the LSG volume density is $3.82\times 10^{22}$ cm$^{-3}$ and the electron density is 6 times more when fully stripped. As discussed above and shown in Supplementary Fig. 2, the composition of the target is proton:oxygen:carbon = 1:0.48:1.24, and that the total electron number, which is essential for the laser-matter interaction, is $(1+0.48\times 8+1.24\times 6)/(1.24\times 6)= 12.28/7.44=1.65$ times more than that from graphene. We fix the electron density as $n_e=1.65\times 6\times 3.82\times 10^{22}=3.78\times 10^{23}$ cm$^{-3}$. Here we consider all the heavier ions are carbon for simplicity; the proton ratio is set to be $1/(1+11.28/6)\sim 0.35$ of all atoms. We define carbon and proton densities as $n_C=0.65\times n_e/(0.65\times 6+0.35)$ and $n_P=0.35\times n_e/4.25$, respectively. Note that we do not consider the graphene structure in the simulations. The numerical parameters used in the runs are settled after the massive conversion tests as $\Delta x=\Delta y=$ 1.6 nm, and $(N_x, N_y)=$ (12,500, 12,500), corresponding to $(L_x, L_y)=(20, 20) \mu$m. Since the target is place 45$^{\circ }$ from the laser propagation axis, which is also along the *x* axis, we have to resolve the target in the *y* direction as well as the *x* direction.

## Supplementary Information

Supplementary Information.

## Data Availability

The data sets generated during and/or analyzed during the current study are available from the corresponding author on reasonable request.

## Code availability

The custom computer code or algorithm used to generate results is available from the corresponding author on reasonable request.
