# Supplementary material for: Robustness of large-area suspended graphene under interaction with intense laser
Source: Sci Rep. 2022 Feb 16;12:2346. doi: 10.1038/s41598-022-06055-4 (PMC8850449; doi:10.1038/s41598-022-06055-4)
Supplement: Supplementary file 1 — Supplementary Information. [file 41598_2022_6055_MOESM1_ESM.pdf]

Supplementary information
Robustness of large-area suspended graphene under interaction with intense laser

Kuramitsu et al.

Thomson parabola spectrometer (TPS)

TPS is one of the most widely used diagnostics for laser driven ion acceleration. By applying electric ($\vec{E}$) and magnetic ($\vec{B}$) fields, TPS allows us to obtain energy spectra for ions with different charge-to-mass ratio. Ions with the same charge-to-mass ratio but different energies form a parabola on a detector plane as schematically shown in Fig. 1 (e). The nominal parameters used in the experiment are $B = 0.165$ T, $E = 0.444$ kV/mm, $L = 100$ mm, and $D = 497$ mm. The diameter of the MCP is 75 mm, and the collimator ($\phi = 300$ μm) is placed ~ 1.5 m away from the target .

Assuming an accelerated ion going along the z-axis with the velocity of $\vec{v}$, and $\vec{E}$ and $\vec{B}$ fields to be in the x-axis, the ion trajectory deviates from the dashed line corresponding to a neutral particle or infinite energy charged particle trajectories due to $q\vec{E}$ and $q\vec{v}\times\vec{B}$ in x and y directions, respectively, in Fig. 1 (e). By neglecting the fringing fields, the displacement in each direction for non-relativistic ions can be written as

$$x_D = \frac{qEL}{mv^2}\left(\frac{L}{2} + D\right), \quad y_D = \frac{qBL}{mv}\left(\frac{L}{2} + D\right), \tag{1}$$

where $q \equiv Ze$ is the ion charge. Eliminating v from Eq. (1), one obtains the parabolic formula on the detector as

$$y_D^2 = \alpha\frac{q}{m}x_D, \tag{2}$$

where $\alpha \equiv B^2L(L/2 + D)/E$.

Supplementary Figure 1 (a) shows the signal from the MCP taken by CCD camera with a commercial lens, which is the same data as in Fig. 4 (a). We fit the proton parabola with Eq. (2) with least squares fitting method using α and the rotation of the image coming from the slight uncertainty of TPS and camera alignment as free parameters. Using the same fit parameters, all the other parabolae with different q/m from carbons and oxygens are well fitted as shown in Supplementary Figure 1 (a). The zero displacement point or infinite energy reference point, which is represented with plus mark in Supplementary Figure 1 (a), is determined from the null target shots. Supplementary Figure 1 (b) shows a TPS image without graphene showing the reference point.

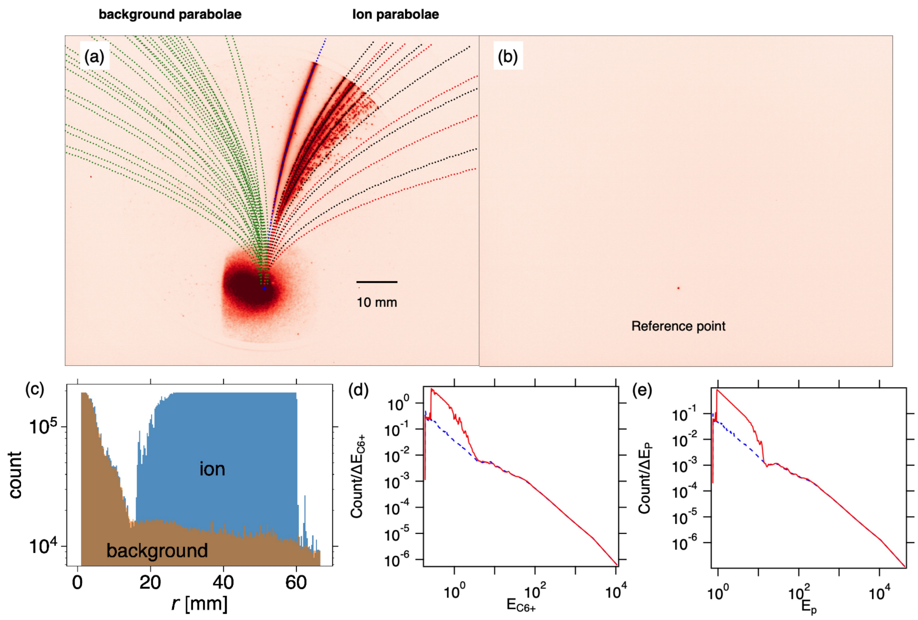

Supplementary Figure 1. (a) Thomson parabola image with 8-layer LSG, which is the same data as in Fig. 4 (a). (b) The reference shot after target shot, i.e., no graphene to obtain the zero displacement point, as depicted plus mark in (a). We consider imaginary particles in order to subtract background from the ion side signals. (c) The background and ion signals in terms of displacement. (d) and (e) The carbon 6+ and proton spectra are shown with the background taken from the symmetric parabolae, respectively.

In order to subtract the background signals, we consider imaginary particles moving oppositely by $\vec{E}$ field but correctly with $\vec{B}$ field as shown in Supplementary Figure 1 (a). Since the background signals come from the x-, γ-rays and also from neutral particles those recombined with electrons on the way to the detector, they are not sensitive to $\vec{E}$ and $\vec{B}$ fields. Since the TPS has mechanical structure symmetric in terms of a line parallel to the vertical axis and going through the reference point, we consider the parabolae corresponding to the imaginary particles as the background in Supplementary Figure 1 (a). For the background parabolae we take a finite width to suppress the spiky noise, which is not very clear in Supplementary Figure 1 (a), but can be seen in Figs. 2 (a) and (b). Supplementary Figure 1 (c) shows the count on the background and ion parabolae as the function of the distance from the reference point. The ion signals at $r \sim 30 - 60$ mm, corresponding to low energy signals, are saturated since we want to observe the high energy region more clearly. By converting the distance to the energy and by dividing the energy bins ΔE, we obtaine

Supplementary Table I. Stopping energies of protons at each front surface except the last of CR-39s [MeV].

	Number of filters					
	0	1	2	3	4	5
1st CR-39	1	4.7	6.7	8.4	10.2	11.5
2nd CR-39	9.6	11.1	12.2	13.2	14.5	15.4
3rd CR-39	14.1	15.1	16	16.9	17.8	18.6
4th CR-39	17.7	18.5	19.2	20	20.9	21.7
5th CR-39	20.8	21.5	22.2	22.9	23.6	24.3
5th CR-39 (back surface)	23.5	24.2	24.6	25.3	26	26.7

the carbon 6+ and proton spectra together with the symmetric parabolae in Supplementary Figures 1 (d) and (e), respectively. The ion and background signals agree very well at high energies, allowing us to estimate the maximum energies of ions. We define the maximum energy when the standard deviation using all the pixels within the energy bin exceeds the mean value. When the signal is weak, the maximum energy can be underestimated.

Stack detector

Stack detector is located along the laser axis with 157 cm from the laser waist to the front surface of the detector. Front surface is covered with Al foil of 12 μm thickness to prevent the direct irradiation of laser on the RCFs and CR-39s, and also to stop a large number of low energy ions. The RCFs are used as a step filter to further resolve the ion energies as schematically shown in Fig. 1 (d). The minimum proton and carbon energies on each layer are summarized in Tables I and II, respectively. The ion stopping energizes are calculated with the PHITS code [1]. Note that the rear side energies on each CR-39s are equal to the next front surface energies, and similarly the maximum energy stopped with a certain number of filters equals to the minimum energy at the next filter. For example, the proton pits observed on the 1st CR-39 with no filter correspond $1 \leq E_p \leq 4.7$ MeV.

Composition

Supplementary Figure 2 (a) shows the same TPS data as in Fig.2 (a), where we add the oxygen parabolae (red dotted lines) for aid of view. As shown in Supplementary Figure 2 (a), not only carbon ions but also oxygen ions are generated from the LSG, although carbons

Supplementary Table II. Stopping energies of carbons at each front surface except the last of CR-39s [MeV].

	Number of filters					
	0	1	2	3	4	5
1st CR-39	14	94	148	185	218	244
2nd CR-39	212	241	268	292	316	337
3rd CR-39	309	333	353	374	392	412
4th CR-39	388	407	426	442	460	476
5th CR-39	456	473	488	503	520	534
5th CR-39 (back surface)	516	531	546	561	574	589

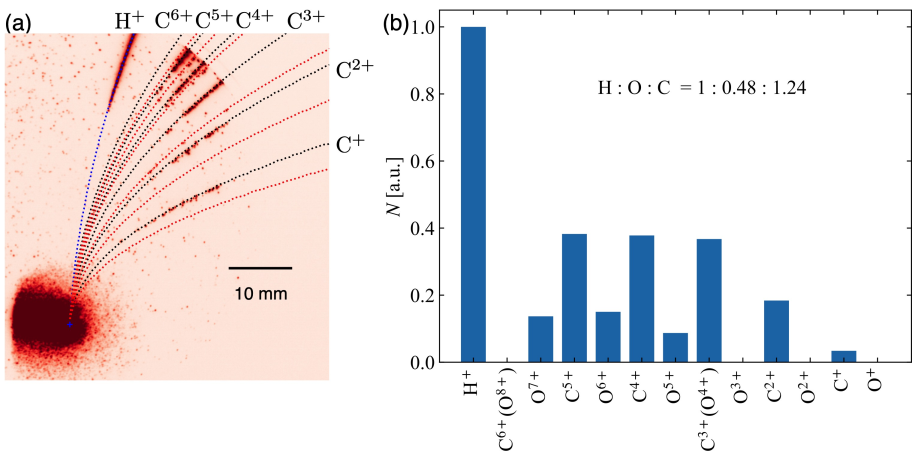

Supplementary Figure 2. **Composition** (a) Thomson parabola image with 4-layer LSG. The dotted lines show the parabolae corresponding to H^{+} (blue), C^{1+}– C^{6+} (black), and O^{1+}– O^{8+} (red), where $O^{8+(4+)}$ lines on the same line as $C^{6+(3+)}$. (b) The composition ratio of LSG generated ions normalized to proton population.

are clearly much more and higher energy than oxygens. Supplementary Figure 2 (b) shows the composition ratio obtained from Supplementary Figure 2 (a). We set the population corresponding to C^{6+}+O^{8+} to be zero. After subtracting the background we normalize all the populations to the proton population. From Supplementary Figure 2 (b) it is evident that the oxygen ions are much less than carbon ions. We sum up carbon and oxygen separately and using the summed ratio we divide the C^{3+} and O^{4+} into carbon and oxygen, and finally

by summing up all the charge states for each ion species we obtained the composition ratio, proton : oxygen : carbon = 1: 0.48 : 1.24. Note that the integral domains for energies are limited by the size of detector, and thus, there are particles outside of our integral domains. Nevertheless, the proton to oxygen is close to two to one, indicating the contamination comes from water. The composition of carbon (H_2O) is 72% (28%). As mentioned above, there is no chemical bond in the Raman spectra other than graphene, and thus, the contaminat should exist as "particles". Note that we also measure the surface contaminant with x-ray photo-spectrometer (XPS) with a radiation facility. Since XPS has to be operated in an ultra-high vacuum chamber ($\sim 10^{-8}$ torr), the chamber condition is different form laser experiment. The XPS measurement shows no proton or oxygen, i.e., the contaminant can be easily removed. It is difficult to keep the target chamber that high vacuum for laser experiment, however, it would be easy to heat up the target during the shots to remove the contaminant. This will be tested in the future experiments.

High contrast defocused shots: thickness dependence.

Supplementary Figures 3 (a)–(h) show the TPS images with different thickness (D) with the defocused shots with the laser energy of $\sim$ 20 J and the intensity of $\sim 10^{18}$ Wcm^{-2} corresponding to $a_0 \sim 0.755 - 0.775$. Note that Supplementary Figure 3 (a) is the same shot as Fig. 2 (c). Supplementary Figures 3 (b) and (c) are both correspond to 12-layer LSG, and result in similar ion energies. Supplementary Figures 3 (f) and (g) are both correspond to 28-layer LSG, however, signal levels are largely different, while the proton energies are similar in both cases. From the TPS images one may see weak dependence on the target thickness. Supplementary Figures 3 (i) shows the maximum ion energy divided by the charge E_i/Z_i against the target thickness. The optimum thickness for the radiation pressure acceleration (RPA) is $1.99 < D < 31.4$ nm [2], which represented with the vertical dotted lines in Supplementary Figure 3 (i). Although the laser is linearly polarized and is different from the ideal condition of circular polarization in [2], our target thickness of 4–32 nm roughly corresponds to the optimum range. Since we estimate the maximum energy once the standard deviation exceeds the mean value within the energy bin (see more details above), the maximum energy for such disconnected signals as Supplementary Figures 3 (g) and (h) may be underestimated. Whether the last pixel is signal or noise can be always issue; C^{6+} signals are relatively weak, and thus, we do not use here C^{6+} to estimate the maximum energies in Supplementary Figure 3 (i). We take average for the same thickness.

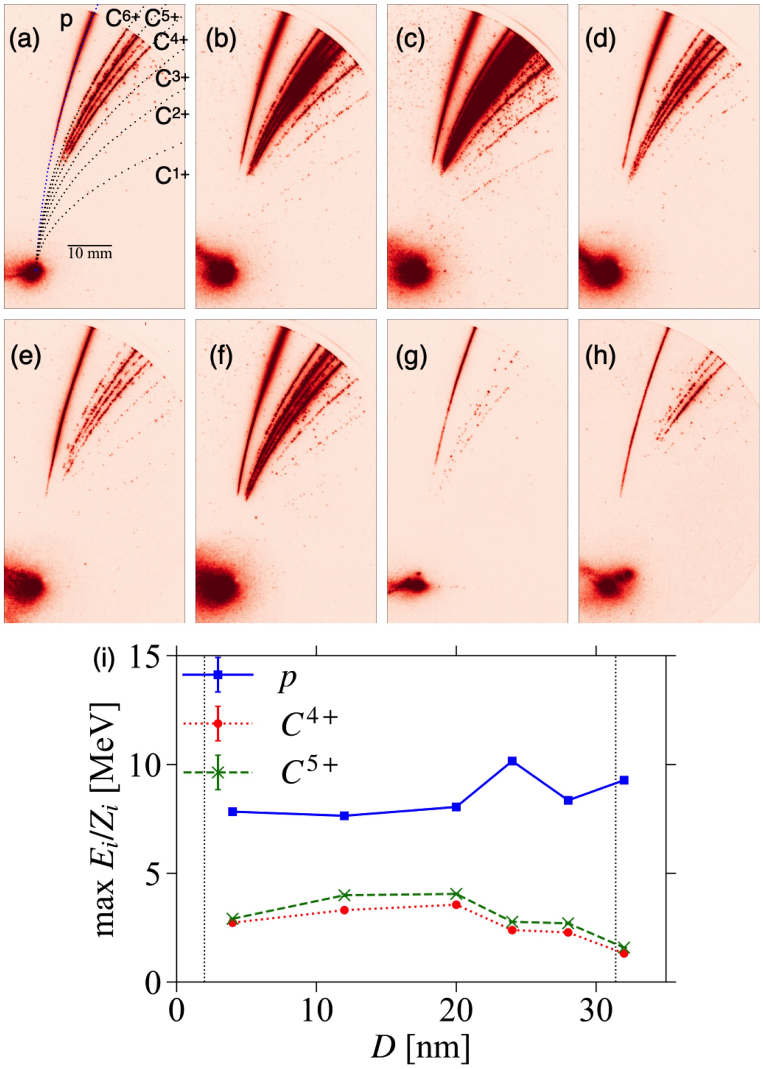

Supplementary Figure 3. **High contrast defocused shots: thickness dependence.** TPS images with increasing target thickness: (a) 4-layer, (b) 12-layer, (c) 12-layer, (d) 20-layer, (e) 24-layer, (f) 28-layer, (g) 28-layer, and (h) 32-layer LSG. (i) The maximum E_i/Z in terms of target thickness. Two dotted vertical lines represent the optimum target thickness for RPA [2].

Even though there are not many shots, the acceleration seems not sensitive to the target thickness in this thickness regime.

Low contrast shots: reproducibility.

In low contrast experiment, the ion acceleration with single-digit-nanometer thick targets result in unstable acceleration, some of them show just proton such as in Fig. 5 (c) with low energies, and some show also carbons with more energies. In order to make the acceleration stable with the low contrast laser, thicker targets are necessary. Supplementary Figure 4

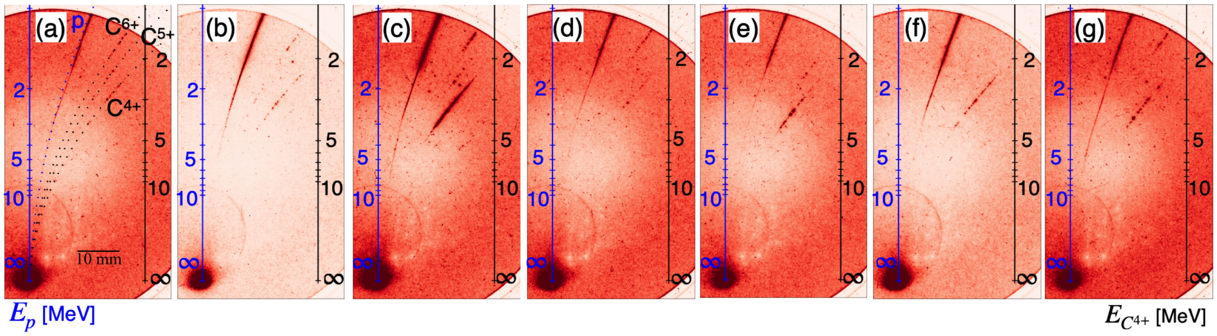

Supplementary Figure 4. **Low contrast shots: reproducibility.** Seven successive target shots with 32-layer LSGs. (a) The laser of 12.1 J, corresponding intensity and normalized intensity of $I = 3.03 \times 10^{21}$ Wcm^{-2} and $a_0 = 37.9$, (b) 14.7 J, $I = 3.56 \times 10^{21}$ Wcm^{-2}, and $a_0 = 41.1$, (c) 14.9 J, $I = 3.73 \times 10^{21}$ Wcm^{-2} and $a_0 = 42.1$, (d) 15.1 J, $I = 3.79 \times 10^{21}$ Wcm^{-2} and $a_0 = 42.4$, (e) 15.0 J, $I = 3.76 \times 10^{21}$ Wcm^{-2} and $a_0 = 42.2$, (f) 14.9 J, $I = 3.73 \times 10^{21}$ Wcm^{-2} and $a_0 = 42.1$, and (g) 14.7 J, $I = 3.68 \times 10^{21}$ Wcm^{-2} and $a_0 = 41.8$. In each panel the left and right vertical scales show the proton and C^{4+} energy.

shows seven sequential target shots with 32-layer LSGs with $a_0 \sim 40$. At this thickness, even with such a large pre-pulse, the LSGs can stably generate MeV protons and carbons. Overall images are similar but one may find the difference in detail; while proton and C^{4+} are almost always generated at similar energies, the C^{6+} and C^{5+} generation depends on the shots. The big hump intensity is 6.6×10^{15} Wcm^{-2} when the peak intensity is 5×10^{21} Wcm^{-2} in Fig. 5 (a). The hump can ionize carbon up to C^{4+} since the threshold intensity for C^{4+} is 4.3×10^{15} Wcm^{-2} as in Fig. 5 (b) [3].

PARTICLE-IN-CELL SIMULATIONS

The results of 2D run with the 45 degrees incidence are shown in Fig. 7. The numerically obtained ion energies are higher than that of experiment, indicating that the target is melted prior to the main laser arrival due to the pre-pulse and pedestal. We consider the effects of pre-pulse and pedestal by using pre-ionized expanded targets. Note that in literatures hydrodynamic code is used to couple with PIC simulations to model the pre-pulse and pedestal. However, a few atomic layer thick target cannot be described by hydrodynamic code; we consider here simple models. Keeping the total number of physical particles con-

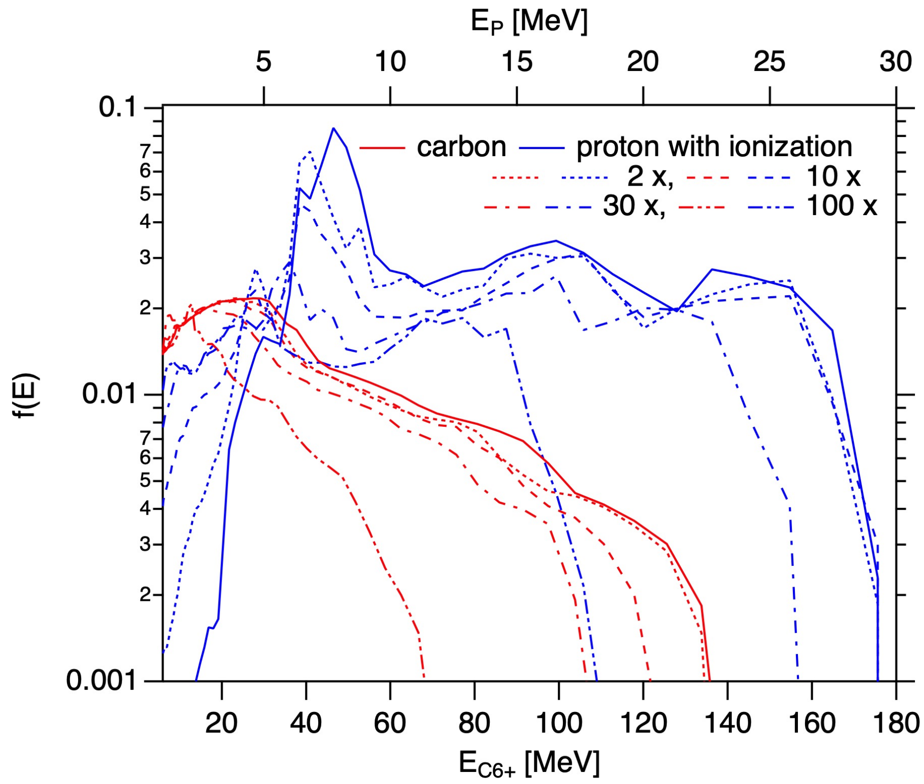

Supplementary Figure 5. The energy distribution functions of carbons (red) and protons (blue) with pre-ionized expanded targets due to the effects of pre-pulse and pedestal of laser. For comparison we also plot the case with ionization with laser (solid lines), which is the same as the solid lines in Fig. 7 (b). We consider the expansions of target thickness by factors 2 (dotted lines), 10 (dashed lines), 30 (dash-dotted lines), and 100 (dash-two-dots lines) and reduce the number density by the same factors. We set the top axis to be 1/6 of bottom axis.

stant, we run the pre-ionized expanded targets, where the thickness is 2, 10, 30, 100 times thicker than the run for Fig. 7 (a), with the reduced densities by the same factors. We also reduce the numerical resolution by multiplying the same factor to Δx and Δy, until it becomes larger than $\lambda/32$, where λ is the laser wavelength. This allows us to save tremendous amount of computation time and resources. Supplementary Figure 5 shows the distribution functions of carbons (red) and protons (blue) where the top axes scale 1/6 of bottom axis. We also show the results with ionization for reference, which is the same as the solid lines in Fig. 7 (b). From these results we can conclude that the ionization is not essential; the twice thicker target with half density (dotted lines) results in almost same distribution functions with ionization (solid lines). As the expansion factor become larger, the carbon and proton

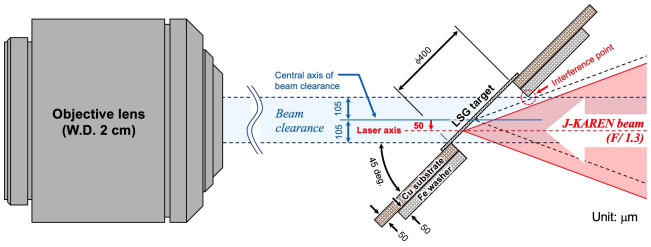

Supplementary Figure 6. The LSG alignment procedure for tight focus short-F laser at 45 degrees incident from the target normal direction.

maximum energies become smaller. Using 100 times thicker target with $0.01n_e$, the carbon and proton energies become comparable to the experimental results. Note that the density is still higher than the critical density.

Alignment procedure

Since the Rayleigh length with the tight focus short-F laser is also very short, we use the objective lens used for the laser focus monitor in order to ensure the target irradiation at the best focus. As shown in Supplementary Figure 6 at the 45 degrees irradiation, if we align the target at the center of the beam clearance, the laser and the target substrate interfere. We move the target as described in Supplementary Figure 6, which is time consuming since the field of view of the focus monitor is also tiny. Thus, the number of shots for the high contrast experiment is limited. We have 16 effective shots in total and all the shots are successful as shown in Figs. 2–4, and Supplementary Figure 3, where MeV protons and carbons are generated, with the high contrast experiment.

For the low contrast experiment, we install an additional monitor for the reflected laser image for safety and we change the incident angle to 10 degrees. We also install three additional target monitors in order to increase the shot rate. In the low contrast experiment with the aid of improved target incident angle and monitoring system, we have many more shots than the high contrast experiments, though we have to use thicker targets to make the acceleration stable.

As shown in Supplementary Figure 6, the ideal beam geometry is already close to the substrate at 45 degrees irradiation. There are always laser energy not in the Gaussian profile. This can interfere the substrate and the x and γ-rays can be generated. The TPS images with the 45-degree irradiation in Figs. 2–4, and Supplementary Figure 3 show asymmetric features around the zero displacement points. The signals around the reference points do not

only come from the neutral particles but also from the x and γ rays from the apparatus. The TPS images with the 10-degree irradiation in Supplementary Figure 4 show more symmetric reference points.

---

[1] T. Sato, Y. Iwamoto, S. Hashimoto, T. Ogawa, T. Furuta, S. ichiro Abe, T. Kai, P.-E. Tsai, N. Matsuda, H. Iwase, N. Shigyo, L. Sihver, and K. Niita, Journal of Nuclear Science and Technology **55**, 684 (2018).

[2] B. Qiao, S. Kar, M. Geissler, P. Gibbon, M. Zepf, and M. Borghesi, Phys. Rev. Lett. **108**, 115002 (2012).

[3] A. Kramida, Y. Ralchenko, J. Reader, and N. A. T. (2018), "Nist atomic spectra database (ver. 5.6.1),".
